# *Bartonella australis* sp. nov. from Kangaroos, Australia

**DOI:** 10.3201/eid1312.060559

**Published:** 2007-12

**Authors:** Pierre-Edouard Fournier, Carmel Taylor, Jean-Marc Rolain, Lina Barrassi, Greg Smith, Didier Raoult

**Affiliations:** * Université de la Méditerranée, Marseille, France; † Queensland Health Scientific Services, Coopers Plains, Queensland, Australia

**Keywords:** *Bartonella*, *australis*, Australia, kangaroo, *Macropus giganteus*, taxonomy, letter

**To the Editor:** During April–May 1999, 3 *Bartonella* isolates (AUST/NH1, AUST/NH2, AUST/NH3) were cultivated and established from the blood of 5 *Macropus giganteus* gray kangaroos from central coastal Queensland, Australia. We used multigene sequencing to evaluate whether these *Bartonella* isolates fulfill the minimum requirements for classification as a new species.

DNA from each *Bartonella* isolate was extracted by using the QIAamp tissue kit (QIAGEN, Hilden, Germany) according to the manufacturer’s instructions. Partial PCR amplification and sequencing of the genes encoding the 16S rDNA (*rrs*), citrate synthase (*gltA*), β-subunit of the RNA polymerase (*rpoB*), and cell division protein (*ftsZ*), as well as for the 16S–23S rDNA intergenic spacer (ITS) were attempted by using previously described primers and conditions (*1*). *Bartonella* sp. isolates AUST/NH1 to AUST/NH3 exhibited identical sequences for all 4 genes and the spacer studied, and isolate AUST/NH1 was selected as type strain among kangaroo isolates. Similarity rates between strain Aust/NH1 and validated *Bartonella* species (Appendix Table) ranged from 84.7% to 91.6%, from 97.5% to 98.5%, from 79.6% to 87.2%, from 85.4% to 95.0%, and from 83.5% to 87.1% for the ITS and *rrs*, *gltA*, *rpoB,* and *ftsZ* genes, respectively. Therefore, for each of these 4 genes or the spacer, strain AUST/NH1 exhibited similarity rates with all other species lower than the cutoffs published to classify *Bartonella* isolates within a validated species (*1*). It may thus be regarded as a new species.

To estimate the genomic G+C content of strain AUST/NH1, we amplified and sequenced its *ftsY* gene as described (*2*) by using the BartftsyF (5′-ATGACAAAAYCYTTTATMAA-3′) and BartftsyR (5′-TCATGAGTGTCTTCCTGC-3′) primers. The *ftsY* G+C content was 37.7%; the calculated genomic G+C content was 39.51%. The *ftsY* sequence was deposited in GenBank under accession no. DQ538398.

The phylogenetic relationships among the studied bartonellae were inferred from sequence alignments of each gene and from concatenated gene sequences by using the maximum parsimony and neighbor-joining methods within the MEGA version 2.1 software package (*3*) and the maximum-likelihood method within the PHYLIP software package (*4*). Using *rrs*, *gltA*, and *rpoB* sequences, the phylogenetic position of strain AUST/NH1 was supported by bootstrap values <70%. In contrast, by using the ITS, *ftsZ,* and concatenated sequences, strain AUST/NH1 clustered with a group of *B. tribocorum*, *B. grahamii*, and *B. elizabethae*, with elevated bootstrap values according to the 3 analysis methods (Figure).

**Figure:**
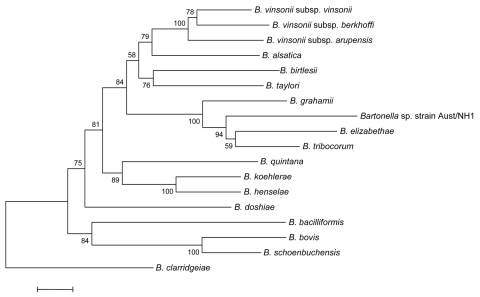
Unrooted dendrogram showing the phylogenetic position of *Bartonella* sp. strain AUST/NH1 among *Bartonella* species inferred from the comparison of concatenated sequences from the *rrs, gltA,* intergenic spacer, *rpoB,* and *ftsZ* genes by the neighbor-joining method. We included only species for which all 5 genes were available. Bootstrap values are indicated at the nodes. The scale bar indicates nucleotide sequence divergence of 0.5%.

The *Bartonella* strains we describe are the first, to our knowledge, obtained from kangaroos and, more generally, from marsupials. Before this study, the only 2 *Bartonella* species found in Australia were *B. henselae* (*5*) and *B. quintana* (*6*). We demonstrated that strain AUST/NH1 was reliably associated with a well-established cluster, including the rodent-associated *B. elizabethae*, *B. grahamii*, and *B. tribocorum* (*7*). Therefore, we are confident that the phylogenic position of the new *Bartonella,* which was similar according to 3 analysis methods and supported by high bootstrap values, is reliable. Although *B. grahamii* (*8*) and *B. elizabethae* (*9*), members of the same phylogenetic cluster as strain AUST/NH1, cause human infections, the pathogenicity of *B. tribocorum* is as yet unknown. Its pathogenicity should therefore be investigated, especially for persons who come in contact with kangaroos.

*B. australis* is a facultative intracellular gram-negative bacterium. It grows on Columbia agar with 5% sheep blood at 32°C to 37°C in a moist atmosphere containing 5% CO_2_. A primary culture was obtained after 7 days, and subculture was obtained after 4 days under the same conditions. Colonies are homogeneous, smooth, round, and gray-white. The 3 strains tested were oxidase negative, catalase negative, and nonmotile. Pathogenicity for humans is, as yet, unknown.

The type strain is strain AUST/NH1. The new species is distinguished from other *Bartonella* species by its 16S rRNA, *gltA*, *rpoB*, *ftsZ* gene sequences, as well as its 16S–23S rRNA ITS sequence. The estimated G+C content is 38%. The type strain exhibits a specific serotype (*10*) and was susceptible to amoxicillin, ceftriaxone, imipenem, erythromycin, clarithromycin, ofloxacin, ciprofloxacin, rifampin, and tetracycline (unpub. data). The type strain AUST/NH1 has been deposited in the Collection of the World Health Organization Collaborative Center for Rickettsioses, Borrelioses and Tick-borne Infections (CSUR), Marseille, France, under reference CSUR B1; in the Collection de l’Institut Pasteur (CIP) under reference CIP 108978T; and in the Culture Collection of the University of Göteborg (CCUG), Sweden, under reference CCUG 51999. The strains AUST/NH2 and AUST/NH3 have been deposited in CSUR under references CSUR B2 and CSUR B3, in the CIP under references CIP 108980 and CIP 108979, and in CCUG under references CCUG 52000 and CCUG 52001, respectively.

## Supplementary Material

Appendix Table Bartonella spp. and sequences used to validate Bartonella isolates (AUST/NH1, AUST/NH2, AUST/NH3) from 5 Macropus giganteus gray kangaroos, Australia, 1999*
