# Supplementary material for: Bartonella australis sp. nov. from Kangaroos, Australia
Source: Emerg Infect Dis. 2007 Dec;13(12):1961–3. doi: 10.3201/eid1312.060559 (PMC2876737; doi:10.3201/eid1312.060559)
Supplement: Appendix Table — Bartonella spp. and sequences used to validate Bartonella isolates (AUST/NH1, AUST/NH2, AUST/NH3) from 5 Macropus giganteus gray kangaroos, Australia, 1999* [file 06-0559_appT-s1.pdf]

Appendix Table. *Bartonella* spp. and sequences used to validate *Bartonella* isolates (AUST/NH1, AUST/NH2, AUST/NH3) from 5 *Macropus giganteus* gray kangaroos, Australia, 1999*

		GenBank accession no.				
Species	Strain	16S rRNA	16S-23S rRNA	*gltA*	*rpoB*	*ftsZ*
B. alsatica	IBS 382^{T}	AJ002139	AF312506	AF204273	AF165987	AF467763
B. bacilliformis	KC584^{T}	Z11683	L26364	U280276	AF165988	AF007266
B. birtlesii	IBS 325^{T}	AF204274	AY116640	AF204272	AF165989	AF467762
B. bovis	91–4^{T}	AF199502	AY116638	AF293394	AF166581	AF467761
B. capreoli	IBS 193^{T}	AF293389	NA	AF293392	NA	NA
B. chomelii	A828^{T}	AY254309	NA	AY254309	NA	NA
B. clarridgeiae	Houston-2^{T}	U64691	AF167989	U84386	AF165990	AF141018
B. doshiae	R18^{T}	Z31351	AJ269786	AF207827	AF165991	AF467754
B. elizabethae	F9251^{T}	L01260	L35103	U28072	AF165992	AF467760
B. grahamii	V2^{T}	Z31349	AJ269785	Z70016	AF165993	AF467753
B. henselae	Houston-1^{T}	M73229	L35101	L38987	AF171070	AF061746
B. koehlerae	C-29^{T}	AF076237	AF312490	AF176091	AY166580	AF467755
B. peromysci		U71322	U77057	NA	NA	NA
B. quintana	Fuller^{T}	M11927	L35100	Z70014	AF165994	AF061747
B. schoenbuchensis	R1^{T}	AJ278187	AY116639	AJ278783	AY167409	AF467765
B. talpae†		NA	NA	NA	NA	NA
B. taylorii	M6^{T}	Z31350	AJ269784	AF191502	AF165995	AF467756
B. tribocorum	IBS 506^{T}	AJ003070	AF312505	AJ005494	AF165996	AF467759
B. vinsonii subsp. *arupensis*	OK 94–513	AF214558	AF312504	AF214557	AY166582	AF467758
B. vinsonii subsp. *berkhoffii*	93-CO1	L35052	AF312503	AF143445	AF165989	AF467764
B. vinsonii subsp. *vinsonii*	Baker^{T}	M73230	L35102	Z70015	AF165997	AF467757
B. australis	AUST/NH1^{T}	DQ538394	DQ538396	DQ538395	DQ538397	DQ538399

*Superscript T, type strain; NA, not available.
†No sequence available for this species.
